# Terminal lipophilization of a unique DNA dodecamer by various nucleolipid headgroups: Their incorporation into artificial lipid bilayers and hydrodynamic properties

**DOI:** 10.3762/bjoc.11.103

**Published:** 2015-06-01

**Authors:** Emma Werz, Helmut Rosemeyer

**Affiliations:** 1 Organic Chemistry I - Bioorganic Chemistry, Institute of Chemistry of New Materials, University of Osnabrück, Barbarastr. 7, 49069 Osnabrück, Germany; 2 Ionovation GmbH, Westerbreite 7, 49078 Osnabrück, Germany

**Keywords:** lipid bilayer, lipo-oligonucleotides, nucleic acids, nucleolipids

## Abstract

A series of six cyanine-5-labeled oligonucleotides (LONs **10**–**15**), each terminally lipophilized with different nucleolipid head groups, were synthesized using the recently prepared phosphoramidites **4b**–**9b**. The insertion of the LONs within an artificial lipid bilayer, composed of 1-palmitoyl-2-oleoyl-*sn*-glycero-3-phosphocholine (POPC) and 1-palmitoyl-2-oleoyl-*sn*-glycero-3-phosphoethanolamine (POPE), was studied by single molecule fluorescence spectroscopy and microscopy with the help of an optically transparent microfluidic sample carrier with perfusion capabilities. The incorporation of the lipo-oligonucleotides into the bilayer was studied with respect to efficiency (maximal bilayer brightness) as well as stability against perfusion (final stable bilayer brightness). Attempts to correlate these parameters with the log *P* values of the corresponding nucleolipid head groups failed, a result which clearly demonstrates that not only the lipophilicity but mainly the chemical structure and topology of the head group is of decisive importance for the optimal interaction of a lipo-oligonucleotide with an artificial lipid bilayer. Moreover, fluorescence half-live and diffusion time values were measured to determine the diffusion coefficients of the lipo-oligonucleotides.

## Introduction

For proper cell function, the biosynthetic lipophilization of proteins and carbohydrates, such as palmitoylation and farnesylation, is of decisive importance [1]. The same seems to be true for bacterial tRNAs [2], which was postulated recently and substantiated by the finding of geranylated tRNAs. Therefore a continuously growing interest in so-called lipo-oligonucleotides (LONs) [3–6] can be observed. In a preceding manuscript [7] we described the base-specific DNA duplex formation at a lipid bilayer–water phase boundary using SYBR Green I staining. For this purpose, various nucleic acids of different length and sequence were lipophilized by appending a terminal non-nucleosidic lipid head group and then bound on the surface of an artificial lipid bilayer. Subsequently, equimolar amounts of complementary and non-complementary DNA strands as well as a SYBR Green I solution were added to the bilayer-bound lipo-oligonucleotide. The kinetics of the ternary complex formation at the bilayer surface as well as the stability of the complexes against perfusion was measured by determination of the bilayer brightness using fluorescence microscopy [8].

In the following, we describe the insertion of six unique single-stranded DNA dodecamers carrying different nucleolipid head groups as well as cyanine-5 (Cy5) at the 5’-(*n*−1) position in artificial lipid bilayers. In this case we varied the amphiphilic head group but preserved the oligonucleotide DNA sequence. This sequence [5’-d(NL-(Cy5) TAG GTC AAT ACT); NL = nucleolipid] was chosen because it forms neither a hairpin structure nor a self-complementary duplex [9]. Particularly, for a successful delivery of siRNAs [10–12] their lipophilization and studies on the interactions of lipophilized nucleic acids with lipid bilayers is of growing importance. As lipophilic head groups we used a series of nucleolipid cyanoethyl phosphoramidites that were recently synthesized [13–17].

## Results and Discussion

**Figure 1:**
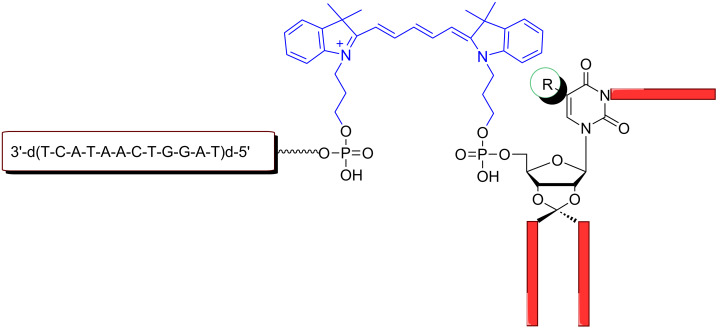
General structure of the fluorophore-labeled lipo-oligonucleotides and positions of lipophilization of the pyrimidine β-D-ribonucleoside head groups **1**–**3** [18].

Figure 1 indicates the positions of the pyrimidine β-D-ribonucleosides uridine (**1**, R = H), 5-methyluridine (**2**, R = CH_3_) and 5-fluorouridine (**3**, R = F) which were lipophilized by different hydrophobic residues.

**Figure 2:**
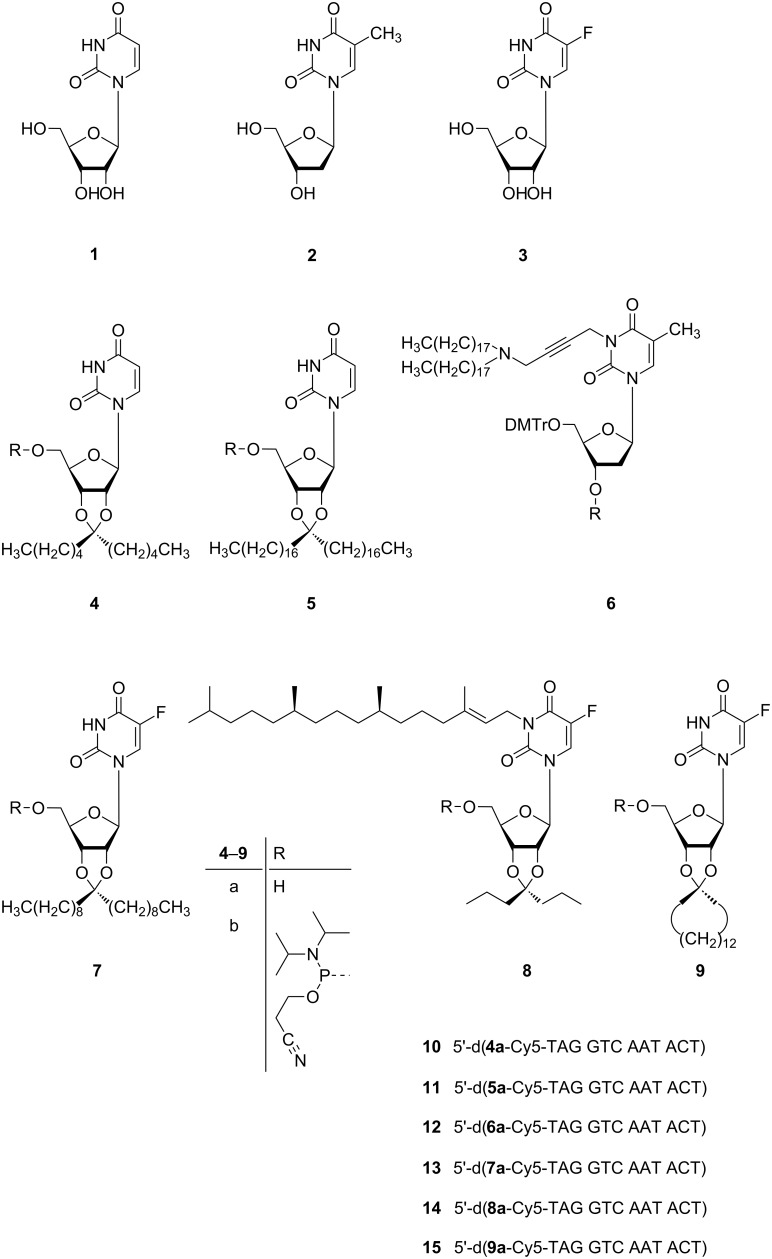
Chemical formulae **1**–**9** and lipo-oligonucleotide sequences **10**–**15**.

The following formulae (Figure 2) show the six nucleolipids **4a**–**9a** [13–17], which designate that mono-, sesqui-, and diterpenes as well as single and double-chained alkyl groups (completed by an alicyclic alkyl group), have been introduced for the lipophilization of the pyrimidine nucleosides. The resulting nucleolipids were converted into their corresponding 2-cyanoethyl phosphoramidites **4b**–**9b**.

**Figure 3:**
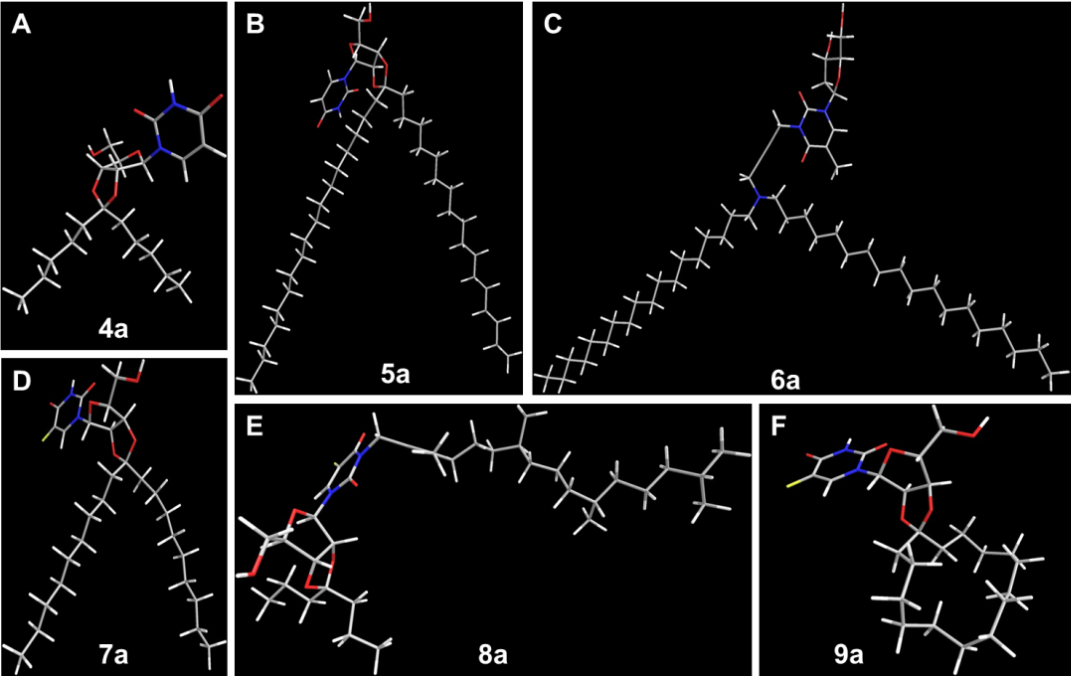
Energy-minimized 3D structures of the lipophilic nucleoside headgroups **4a**–**9a**. All 3D structures were calculated using the program ChemBio3D Ultra v. 12.0 (number of iterations, 387 ± 147, standard deviation of the mean).

Figure 3 shows the energy-minimized 3D structures of the lipophilic nucleoside headgroups **4a–9a**.

The reactive building blocks **4b–9b** were subsequently used to synthesize the following lipo-oligonucleotides (LONs), which have an identical nucleotide sequence in common as well as a cyanine-5 (Cy5) fluorophore at the (*n*−1) position. The integrity of all oligonucleotides was proven by MALDI–TOF mass spectrometry (see Experimental).

### Insertion of the six LONs into an artificial lipid bilayer and their specific characteristics

**Figure 4:**
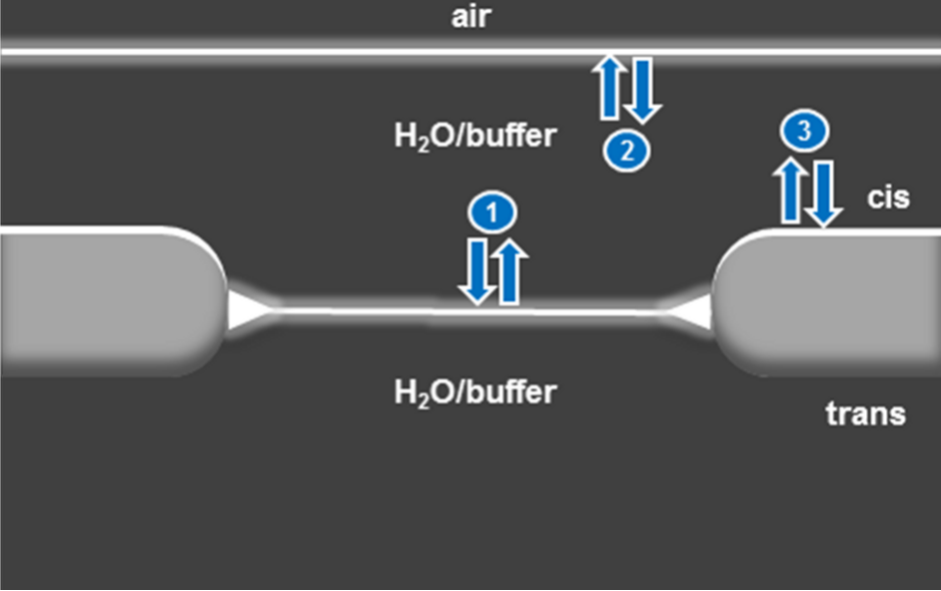
Schematic display of the three main equilibria existing on the cis side of the bilayer chamber.

The different lipo-oligonucleotides (LON **10**–**15**) were incorporated into stable artificial lipid bilayers, which create two compartments (cis/trans channel) of an optically transparent microfluidic sample carrier with perfusion capabilities, as shown in Figure 4. Both the cis and the trans channel were filled with saline buffered solution. The injection of the different oligonucleotides into the cis channel followed by cycles of incubation and perfusion leads to the binding of the corresponding oligomer on the membrane, as detected by microscopy and single molecule fluorescence spectroscopy. The kinetics of the binding of the different bilayer-bound oligonucleotides were studied as well as their stability against perfusion.

Figure 4 illustrates the three main equilibria: the lipid bilayer–H_2_O boundary, the air–H_2_O interface boundary, and at the Teflon surface, which exist on the cis side of the bilayer chamber. This was proven experimentally as shown later in Figure 9.

The technique and some of the results are visualized in a video which can be downloaded free from the repository (RepOSitorium) of the library of the University of Osnabrück, Germany [18].

**Figure 5:**
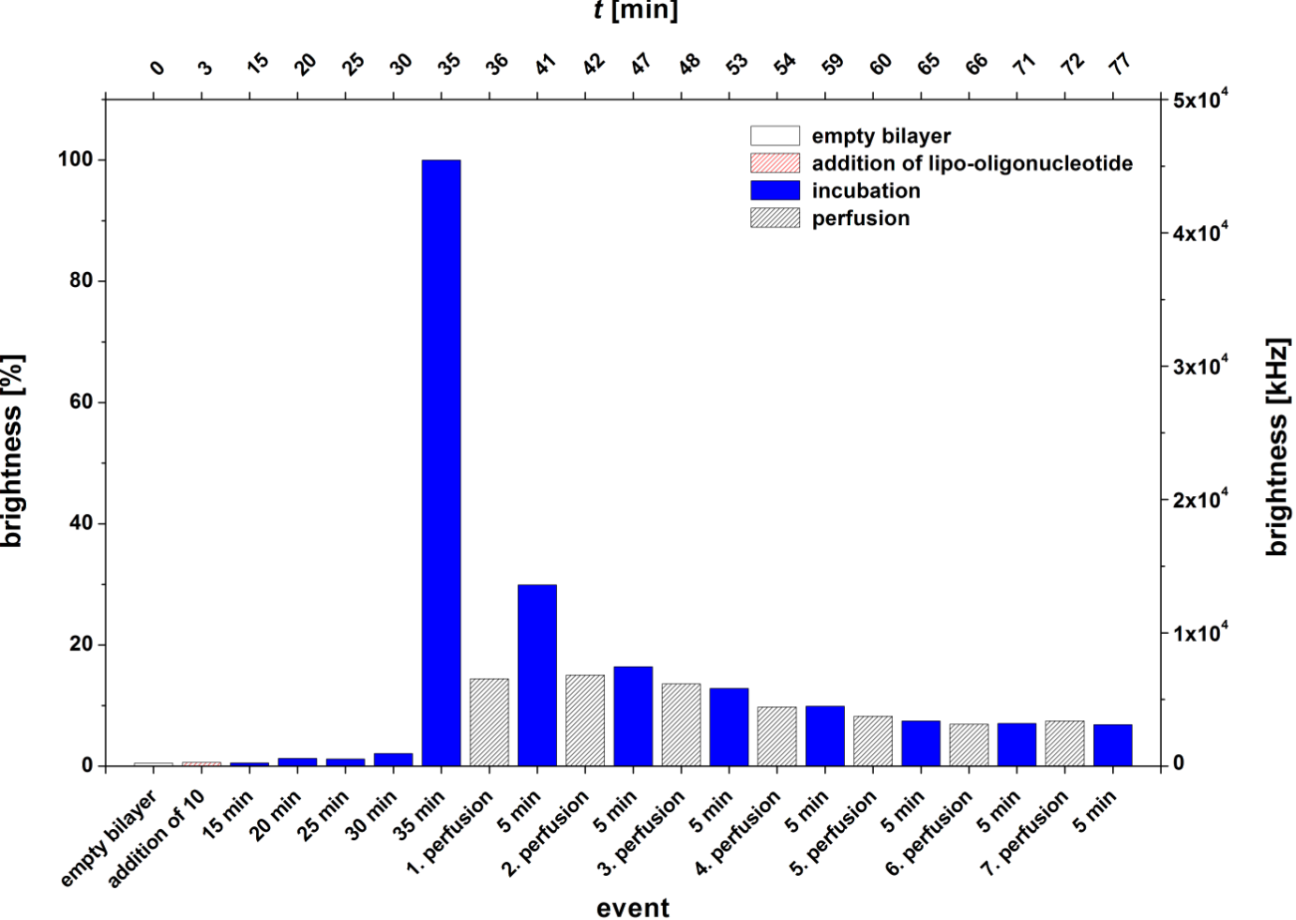
Bilayer brightness vs event graph of 5’-d(**4a**-Cy5-TAG GTC AAT ACT) (**10**).

Figure 5 displays the bilayer brightness (both normalized to 100% as maximum as well as in kHz) as a function of the various events (perfusion and incubation time) for the lipo-oligomer **10**.

As can be seen, the incorporation of the lipo-oligomer **10** carrying an *O*-2’,3’-undecylidene-modified uridine head group into the artificial lipid bilayer occurs spontaneously after 25 min, corresponding to the maximum brightness. However, even after a single perfusion, the brightness is reduced to about 15%, and after further incubation, it is again enhanced to 30% brightness. Subsequent perfusion and incubation periods over the course of roughly 1 h resulted in a constant brightness of 5–10%.

**Figure 6:**
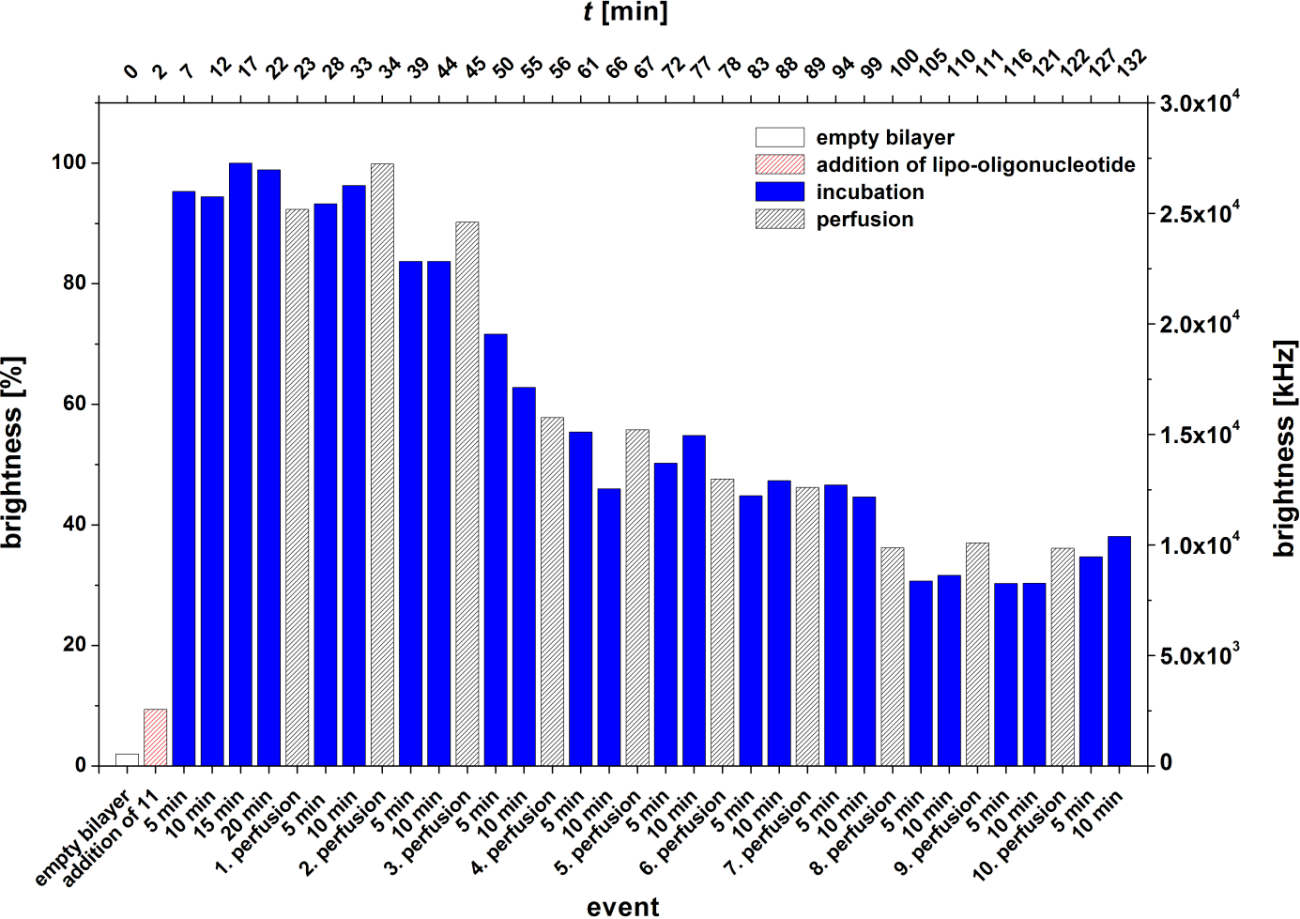
Bilayer brightness vs event graph of 5’-d(**5a**-Cy5-TAG GTC AAT ACT) (**11**).

In contrast to this, an *O*-2’,3’-pentatriacontanylidene-modified uridine head group shows a different behavior (Figure 6): the corresponding lipo-oligonucleotide **11** is bound directly after addition into the cis compartment of the sample carrier within the lipid bilayer (5 min). Once bound it exhibits a significantly higher resistance against perfusion of the cis chamber, and after approximately 100 min (8 perfusions), a constant bilayer brightness (40%, 1 × 10^4^ kHz) is reached. This demonstrates the significant influence of the *O*-2’,3’-alkylidene chain length of the nucleolipid head group on the stability of the lipo-oligonucleotide within the lipid bilayer against perfusion.

**Figure 7:**
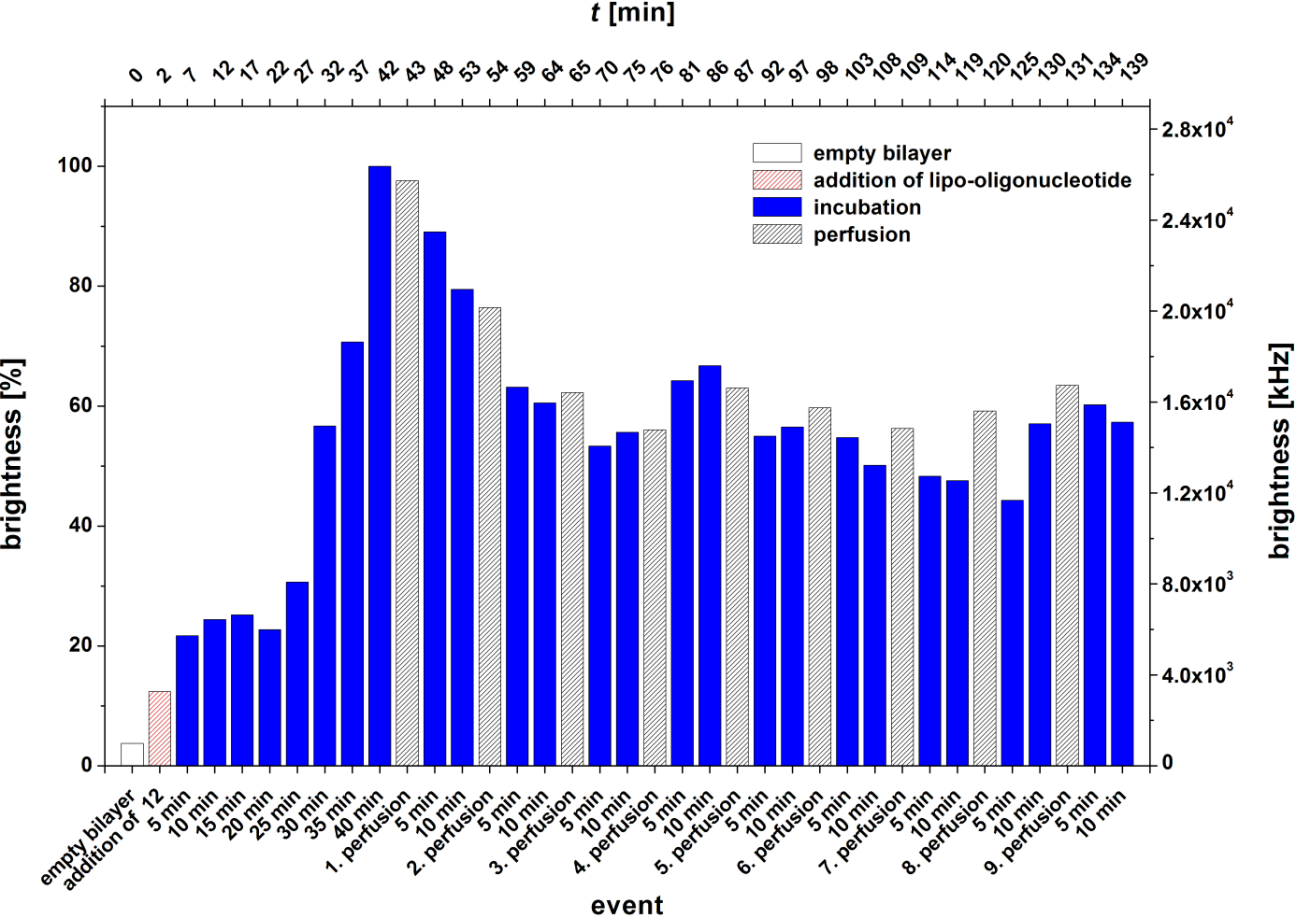
Bilayer brightness vs event graph of 5’-d(**6a**-Cy5-TAG GTC AAT ACT) (**12**).

Figure 7 presents the bilayer brightness as a function of the perfusion/incubation events for the oligomer **12**. This oligomer is 5’-terminated with a nucleolipid head group which carries a double-chained lipid with a clickable group at N(3) of thymidine (**6a**). Among all nucleolipid head groups, this is the only one which can be incorporated once or more at each position of a growing oligonucleotide chain. As was found for the oligomer **10** (Figure 5), a maximum brightness was not immediately reached, but rather after a long incubation time (42 min). However, in contrast to the aforementioned experiment, the oligomer **12** is highly resistant against perfusions. After 3 perfusions, interrupted by incubation periods of 10 min each, a constant normalized bilayer brightness of about 60–65% is reached, which stays constant for more than 1 h and 6 perfusions.

**Figure 8:**
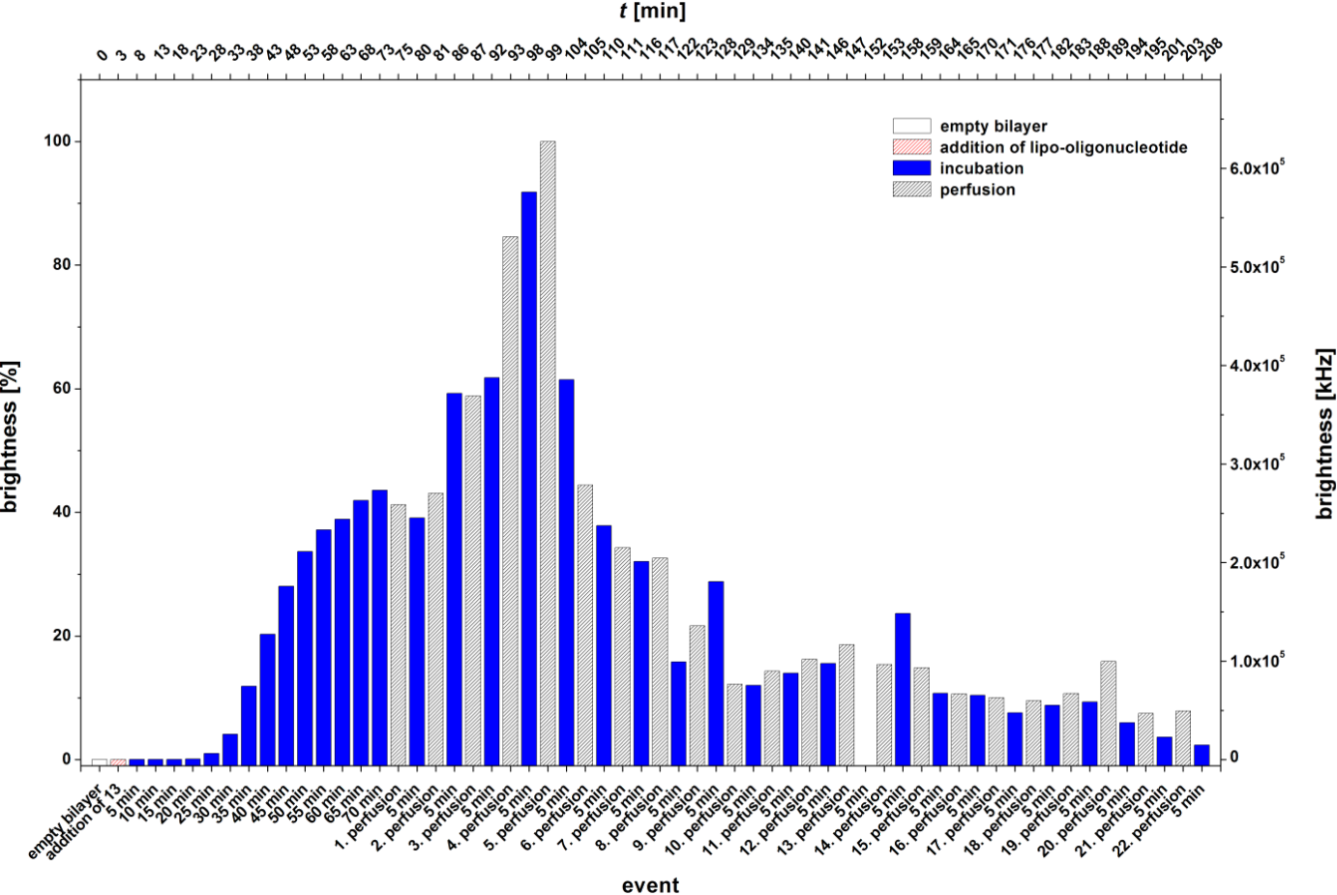
Bilayer brightness vs event graph of 5’-d(**7a**-Cy5-TAG GTC AAT ACT) (**13**).

**Figure 9:**
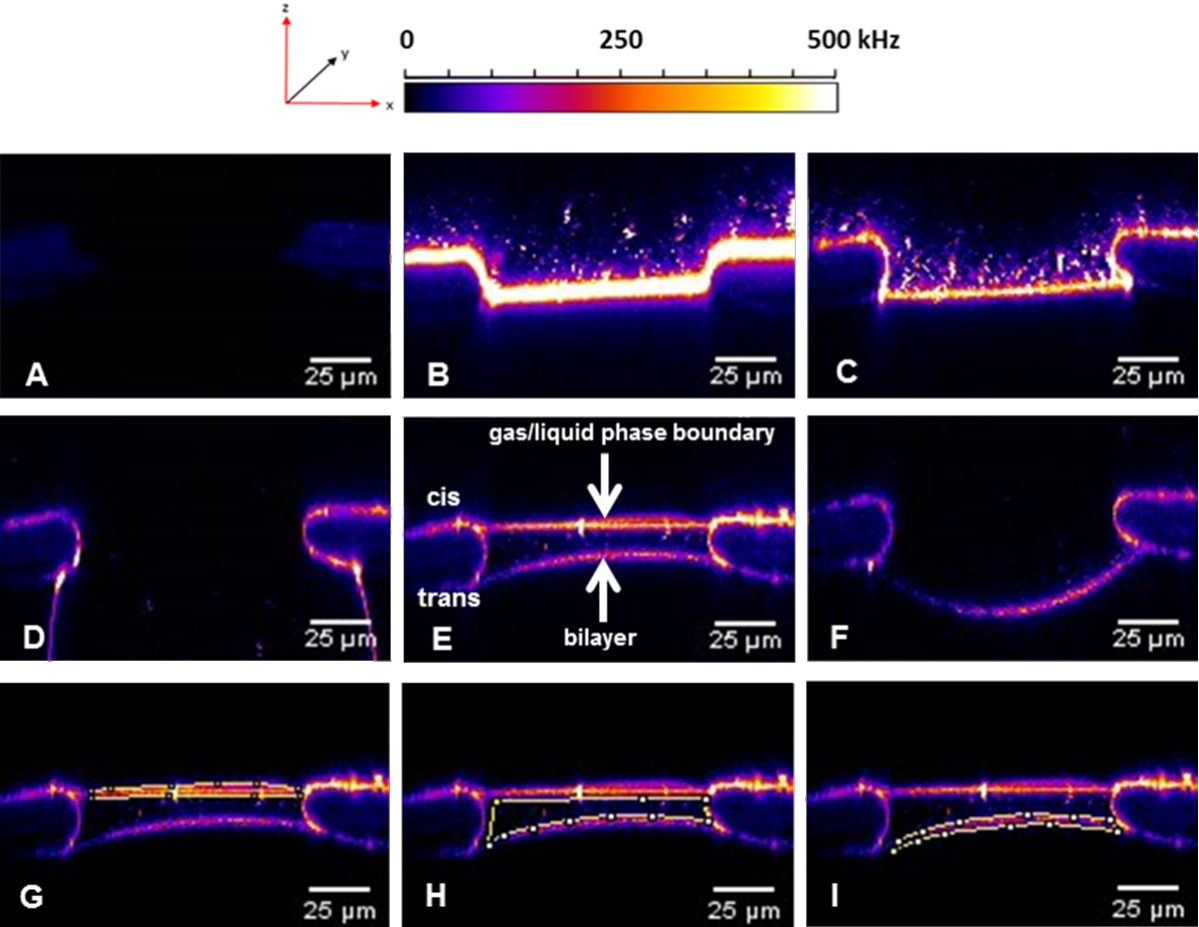
Infiltration of lipo-oligonucleotide (**13**) aggregates from the buffer into the lipid bilayer. Z-direction scan A) of a pure lipid bilayer without fluorescence, B) after the fourth perfusion/incubation step with high bilayer fluorescence and an accumulation of fluorescent aggregates/micelles above the bilayer. C) Z-direction scan of the bilayer after the ninth perfusion/incubation step, resulting in decreased bilayer brightness and an increased amount of micelles. D) After the 13th perfusion/incubation step, the bilayer sags into the trans compartment. After the 14th perfusion the bilayer returns to the starting position. E) After the 22nd perfusion/incubation step, the air–buffer boundary of the cis compartment was lowered over the bilayer. F) Z-direction scan after raising the buffer out of the focus. Brightness of the air–buffer boundary and of the lipid bilayer, each determined within the region of interest. G) The brightness of the liquid–air boundary is approximately 100,000 kHz. H) The brightness of the area between the two boundaries is approximately 28,000 kHz, and the brightness of the bilayer is approximately 50,000 kHz. I) The ratio of the brightness values of the liquid–air boundary and that of the bilayer is 2.

Figure 8 displays the adsorption and desorption results for the lipo-oligonucleotide **13** carrying an *O*-2’,3’-nonadecylidene-lipophilized 5-fluorouridine at the 5’-terminus. This oligomer shows a somewhat unusual behavior upon binding on the lipid bilayer. After 70 min the bilayer brightness reaches a first maximum at about 45%, which stays constant for about 10 min, even during 2 perfusions. Subsequently, however, between *t* = 81 min and *t* = 99 min, the bilayer brightness significantly increases from 40 to 100% (third to fifth perfusion). Further perfusions (up to the 22nd) and incubation periods (until *t* = 208 min) lead to an exponential decrease of the brightness value to almost zero. In order to study this further, the level of the buffer phase in the cis compartment was lowered until it could be visualized within the focus of the fluorescence microscope (Figure 9).

From the z-direction scan shown in Figure 9E (for which the air–buffer boundary was lowered until it could be observed within the focus), it can be recognized that the lipo-oligonucleotide is concentrated at various phase boundaries: (1) at the lipid bilayer–H_2_O boundary, (2) at the air–H_2_O phase boundary, as well as (3) at the Teflon surface. Therefore, the further enhancement of the bilayer brightness (as in Figure 4 at *t* = 81 min) might be due to a redelivery of the lipo-oligonucleotide from (2) or (3).

Transport of the oligomer through the aqueous phase most likely occurs in the form of micelles. For example, the infiltration of such micelles was demonstrated after the 9th perfusion step (Figure 4). Moreover, from Figure 9E it can be seen that the bilayer is significantly arched upwards towards the trans chamber so that it is out of focus. This indicates the development of a Donnan pressure caused by the existence of significantly different osmotic pressures within the cis and the trans compartment, which is due to the fact that the polyanionic nucleic acid is unable to cross the artificial bilayer.

**Figure 10:**
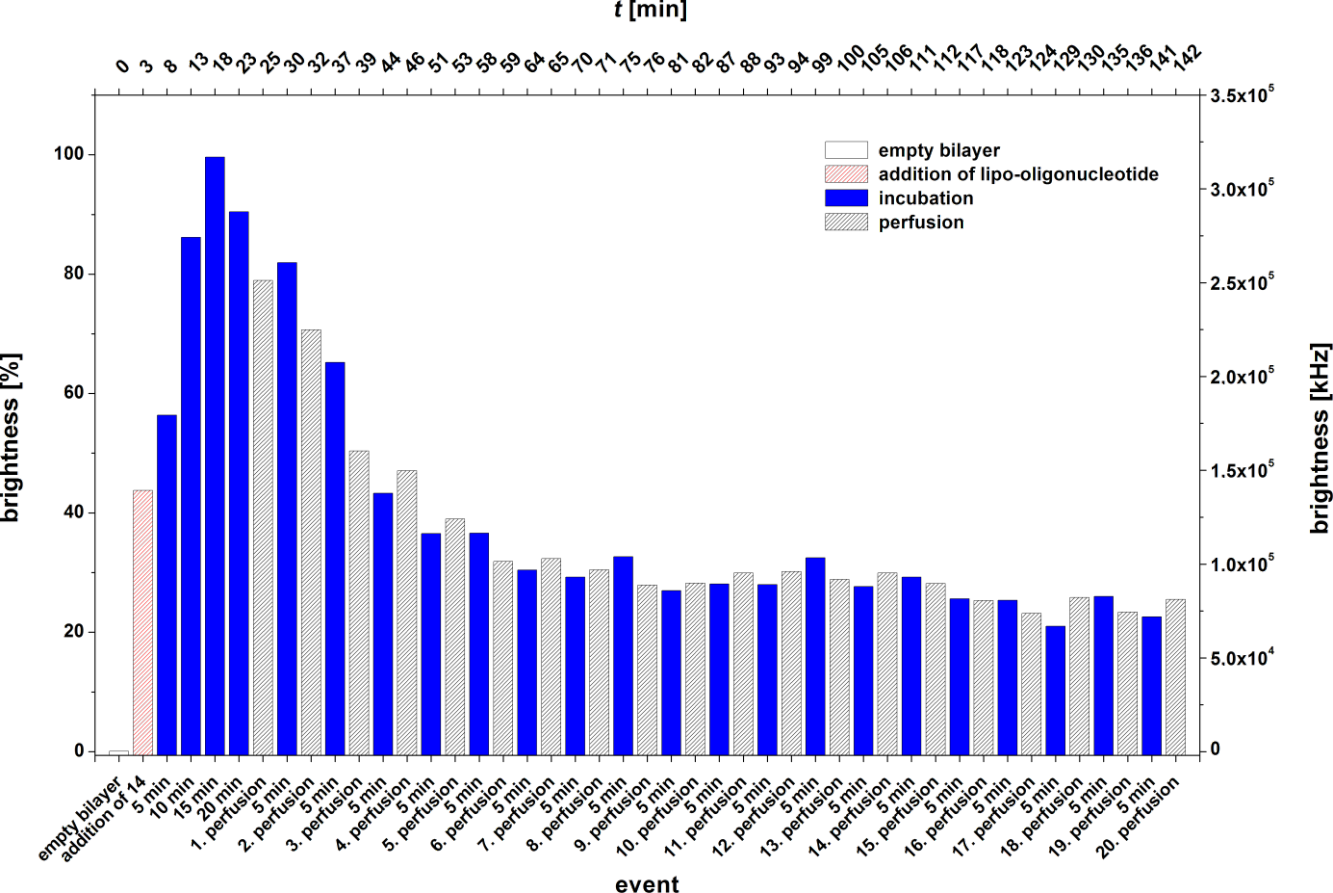
Bilayer brightness vs event graph of 5’-d(**8a**-Cy5-TAG GTC AAT ACT) (**14**).

Figure 10 shows the bilayer brightness vs the various events for the lipo-oligonucleotide **14**. This oligomer carries a phytyl residue at the N(3) atom of 5-fluorouridine (**8a**). Nature uses this diterpenoid group for a membrane binding of chlorophyll, tocopherol and other biomolecules.

The lipo-oligonucleotide is characterized by being very quickly bound at the lipid bilayer. After 15 min the maximum brightness is reached followed by an exponential decrease over the course of six perfusion/incubation periods. A stable bilayer brightness is reached after approximately 1 h after, which remains constant for at least another 90 min.

**Figure 11:**
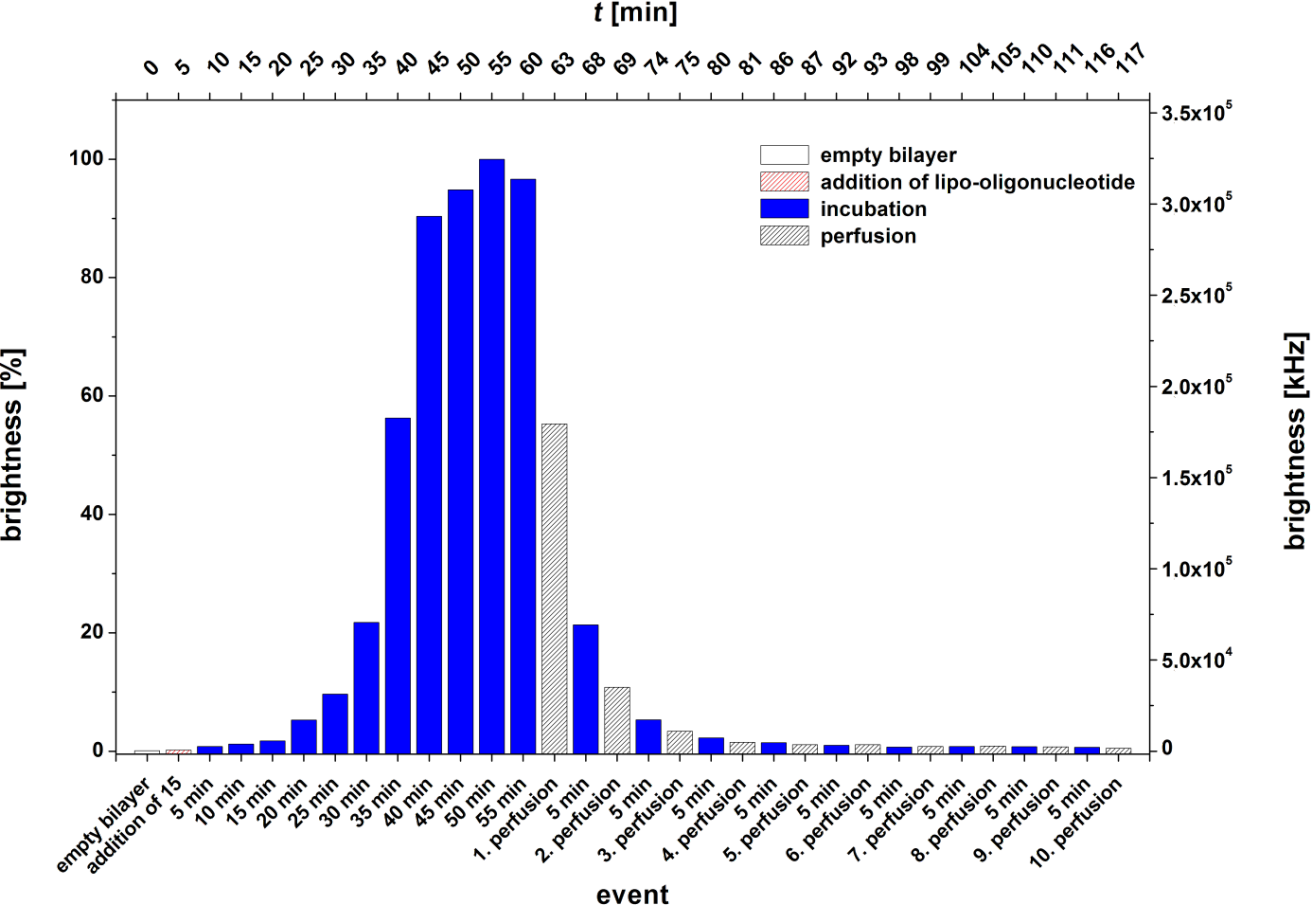
Bilayer brightness vs event graph of 5’-d(**9a**-Cy5-TAG GTC AAT ACT) (**15**).

The incorporation kinetics and stability of the lipo-oligonucleotide **15** are shown in Figure 11. As the nucleolipid head group, it contains 5-fluorouridine carrying an alicyclic *O*-2’,3’-cyclopentadecanylidene ring. As can be seen, the binding within or (more likely) at the lipid bilayer occurs relatively slowly within 55–60 min until the maximum brightness of the bilayer is reached. A single perfusion reduces the brightness to 55%. Further perfusion, followed by incubation periods of 5 min, leads to an almost complete removal of the lipo-oligonucleotide (*t* ≤ 80 min). We therefore assume that the lipophilic moiety of the head group is too bulky (Figure 3F) to be fully inserted into the bilayer and that the lipo-oligonucleotide is only loosely adsorbed at the bilayer surface.

From these results, it can be stated that the insertion rate of a lipo-oligonucleotide into an artificial lipid bilayer as well as its stability within the bilayer does not exclusively depend on the lipophilicity of the head group but also (i) results from the three-dimensional structure of the latter, (ii) from the length of the lipophilic moiety and (iii) from the position of the lipid residue at the nucleoside.

**Figure 12:**
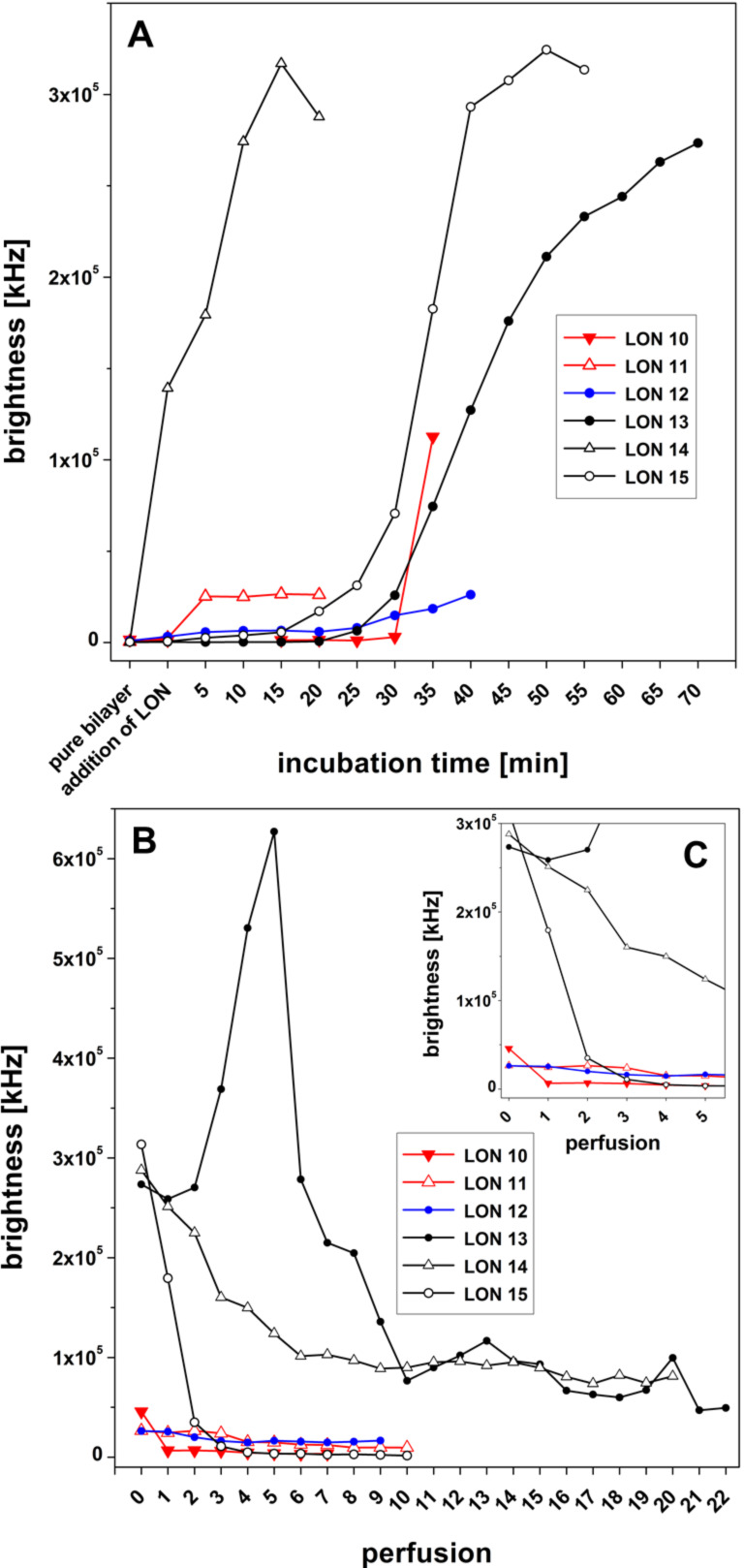
Summarizing the bilayer brightness for LONs **10–15** depending on the incubation time up to the maximum brightness (A), as well as on the number of perfusions (B) with details shown in (C).

For the LONs **10** and **11** we observed that the elongation of the C-atom chain length from 5 to 17 does not necessarily lead to an enhancement of the amount of the LON incorporated into the bilayer. LON **10** is on one hand side incorporated very fast and in a high amount, and on the other hand, 85–90% is released from the bilayer very easily by just a single perfusion (Figure 12A,B). In contrast, LON **11**, which differs from LON **10** only in the C-atom chain length of the lipid moiety, is incorporated only at a relatively low amount but stays bound to a significantly greater extent (40–50%), even after 10 perfusions (Figure 6).

Other lipophilic head groups such as LONs **13**–**15** lead to a fast and efficient incorporation into the bilayer (Figure 12A). Here, we observed LON **14** carrying a single-stranded phytyl residue at N(3) of 5-fluorouridine plus a short *O*-2’,3’-ketal group. This was also observed for LON **13** with an *O*-2’,3’-nonadecylidene ketal group which had a very strong binding within the bilayer, much stronger than that observed for LON **15** with a cyclic *O*-2’,3’-ketal, which was washed out completely from the bilayer after only three perfusion steps (Figure 11).

LON **12** represents an oligomer with a long double-tailed lipid moiety at the N(3) position of thymidine. Interestingly, this LON is incorporated into the bilayer at a relatively low rate but proved highly stable against perfusion.

### Diffusion time measurements of LON 10 inserted within the lipid bilayer

**Figure 13:**
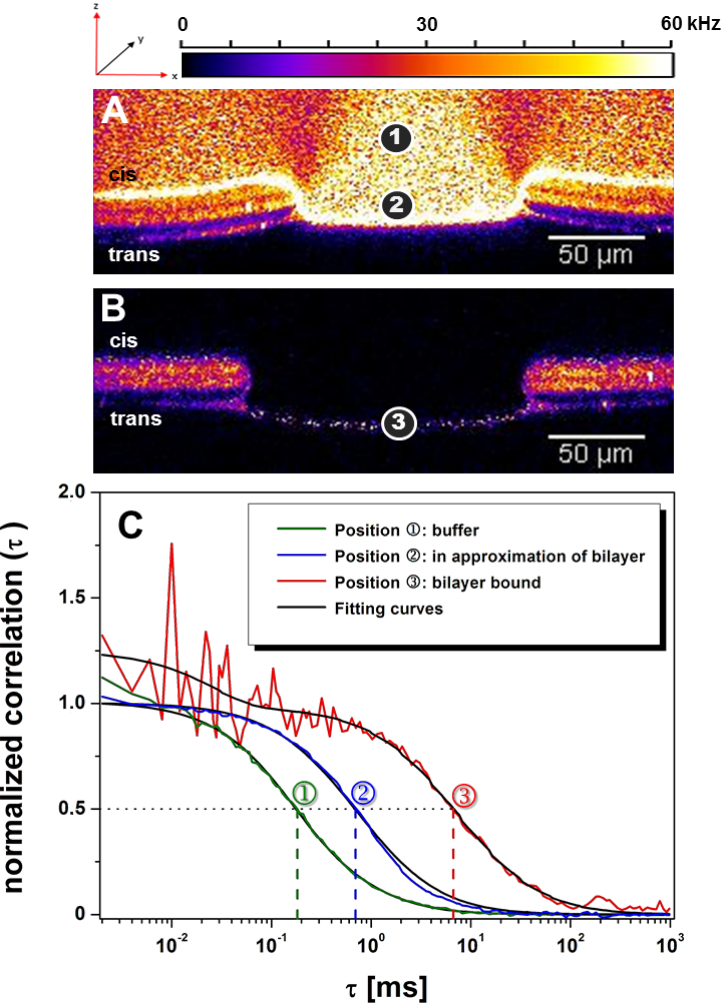
Diffusion time measurements of LON **10** (5 µL, 50 nM). A) Z-direction scan directly after the addition of the oligomer probe into the cis compartment of the bilayer chip. B) Z-direction scan after 7 perfusion/incubation cycles upon which unbound oligomer was completely washed out. This allows a diffusion time measurement of bilayer-bound LON **10**. C) Normalized and fitted correlation curves of the measured diffusion times at positions 1, 2 and 3.

Additionally, the diffusion times of LON **10** at positions 1, 2, and 3 (Figure 13A–C, Table 1) were measured in order to characterize its binding at and within the bilayer. Right above the bilayer (position 2), the diffusion of the oligomer is significantly reduced as compared to that in free solution (position 1).

**Table 1:** Diffusion times, τ_D_ (ms), of LON **10** in the presence of a lipid bilayer. Position: 1 - buffer; 2 - solution in close proximity to the bilayer; 3 - bilayer-bound.

Position		τ_D_ [ms]

**1**	in buffer	0.25 ± 0.08
**2**	close to the bilayer	0.73 ± 0.01
**3**	bilayer-bound	4.25 ± 0.98

From the fitted and normalized fluorescence correlation curves (Figure 13C), the corresponding diffusion times could be deduced as summarized in Table 1. The decreasing mobility of the oligomer from position 1 < position 2 < position 3 clearly indicates a loose adsorption at the bilayer (position 2) followed by incorporation within the bilayer (position 3).

Obviously, at position 2 (which is only ≈5–10 µm away from the bilayer), an attractive interaction exists between a LON and the lipids of the bilayer, which leads to a decrease in diffusion time. However, it is not clear if the diffusion slows down gradually. For this, it would be necessary to measure the diffusion at various positions above or below the bilayer. This is, however, difficult because of the floating of the bilayer.

### Correlation between the structure of the lipophilic head groups of the lipo-oligonucleotides and their stability within the artificial lipid bilayer

Next, we tried to correlate various parameters taken from the “brightness vs event” graphs such as the absolute maximum bilayer brightness values (in MHz) as well as the final stable brightness data (in MHz and %) with the log *P* values of the corresponding nucleolipid headgroup using Tetko’s ALOGPS method (v. 3.01) for the calculation (Table 2). As can be seen in Table 2, no obvious correlation could be deduced. This means that the lipophilicity of the nucleolipid head group is of minor importance for the effectiveness of lipo-oligonucleotide binding within the artificial lipid bilayer with respect to (i) the maximal bilayer brightness, (ii) the binding rate to reach this maximum brightness, (iii) the stability of the LON within the bilayer (expressed in number of perfusions to reach a stable brightness, and (iv) the final constant bilayer brightness. The log *P* values can only be correlated with the number of C-atoms of the lipid moieties, which are probably responsible for the binding to the bilayer. Of decisive importance for an effective insertion of a LON is obviously the chemical structure and topology of the corresponding nucleolipid head group. An acceptable compromise seems to be the phytol-labeled 5-fluorouridine head group, which has a mean log *P* value (6.43 ± 0.74) of the nucleolipid. The corresponding LON **14** reaches a high maximum bilayer brightness (316.9 MHz) within 15 min and proves also to be relatively stable within the bilayer (final brightness, 88.5 MHz). The log *P* values of the nucleotides uridine, 2-deoxythymidine and 5-fluorouridine are as follows: −1.90, −0.96 and −1.30 (±0.38 with a confidence interval of 66%). Thus they prove significantly smaller than the value of the corresponding lipo-oligonucleotides. The log *P* data gives insight into the augmentation of the lipophilicity of the nucleosides **4a–9a**.

**Table 2:** Experimental results of the incorporation of the LONs into artificial lipid bilayers with respect to the log *P* values of the nucleolipid head groups.

	LON					
	**10**	**11**	**12**	**13**	**14**	**15**

Nucleolipid head group (number of C-atoms of the lipid moieties)	**4a** (11)	**5a** (35)	**6a** (36)	**7a** (19)	**8**a (20)	**9a** (15)
log *P*^a^ of head groups	2.27^b^	9.07^b^	9.04^b^	5.34^b^	6.43^b^	3.99^b^
Maximum bilayer brightness [MHz]^c^	45.77	26.52	26.22	273.49 (sh)/ 627.14^d^	316.97	324.46
Time to reach maximum brightness [min]	35	15	40	70^e^/ 99^f^	15	55
Number of perfusion steps to reach a stable bilayer brightness	4	7	3	1/ 16	6	4
Final stable bilayer brightness [MHz]	3.64 ± 0.56	9.78 ± 1.60	14.87 ± 1.52	258.16 ± 1.52/ 53.95 ± 20.68	88.51 ± 9.71	2.98 ± 0.99
Final normalized stable bilayer brightness [%]	7.96 ± 1.22	36.88 ± 6.04	56.73 ± 5.80	41.16 ± 2.00/ 8.60 ± 3.00	27.93 ± 3.06	0.92 ± 0.31

^a^log *P* values were calculated at the http://eadmet.com/de/physprop.php site with ePhysChem that uses ALOGPS v. 3.01 for log *P* prediction. ^b^±0.74 with a confidence interval of 66%. ^c^The brightness of the empty bilayer amounts to 425 ± 300 kHz (*N* = 6). ^d^Reached after 5 perfusions. ^e^First plateau, reached after 1 perfusion. ^f^Constant final brightness, reached after 16 perfusions.

### Calculations and experimental measurements of hydrodynamic properties of lipo-oligonucleotides

#### Simulation and modeling of the LONs and calculation of their hydrodynamic parameters

**Figure 14:**
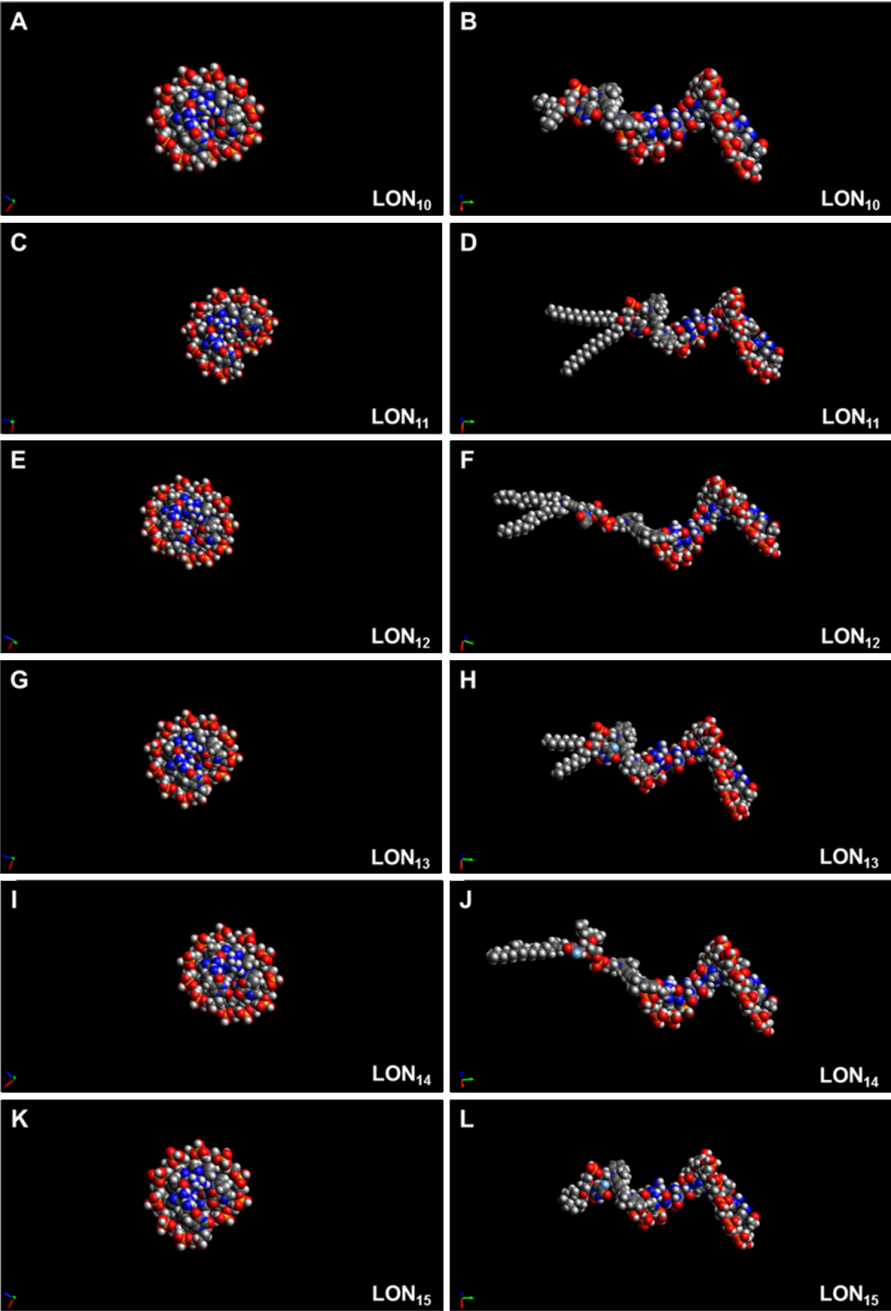
Hydrodynamic models of lipophilic oligonucleotides **10**–**15**. Views are perpendicular (right) or parallel (left) to the helix axis.

For the calculation of the hydrodynamic properties of LONs **10**–**15**, first the 3D structure of the single-stranded oligomer sequence 5’-d(TAG GTC AAT ACT)-3’ was calculated using the HyperChem software (Hypercube, ON, Canada) and then 5’-linked to the structures of the lipophilic head groups. The resulting 3D structures of the LONs were subsequently energy-minimized by applying the MMFF94 force field by using the freely available software package, Avogadro v 1.1.1 [19] (Figure 14).

**Figure 15:**
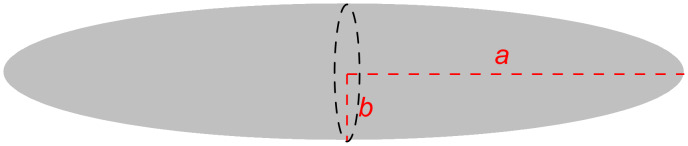
Scheme of a stretched rotation ellipsoid with the semi-major axes *a* and *b*.

For the subsequent calculations of the viscous drag and the diffusion coefficient, *D,* LONs **10**–**15** were adapted to the form of a stretched rotation ellipsoid, moving at random (Figure 15). For this purpose, from the 3D structures (Figure 14) of the LONs, the half axes *a* and *b* were deduced. The half axis *a* amounts to half of the sum *a*_1_ – *a*_3_ (Figure 15, Equation 1). The half axis *b* is half of the diameter of the DNA single strand of the LON and is approximated using Pythagoras’ theorem. For this assessment, the distances between the phosphate groups P_1_ and P_5_, P_5_ and P_9_ as well as between P_9_ and P_1_ were taken from the 3D structures from the 12-mer DNA sequence using the Avogadro or the RasMol software. Half of the diameter corresponds to the half axis, *b*.

Because nucleic acids bind water molecules and are, therefore, covered by a hydration shell, 2.8 Å is added to the radius *b* which is the diameter of a water molecule. The mean value of the semi-minor axis, *b*, of a hydrated nucleic acid single strand is 12.03 ± 0.15 Å.

The frictional coefficient, *f*, of an ellipsoid, moving at random, with the semi-major axis *a* and semi-minor axis *b* [20], is described by Equation 2:

[2]
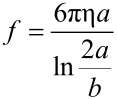


**Figure 16:**
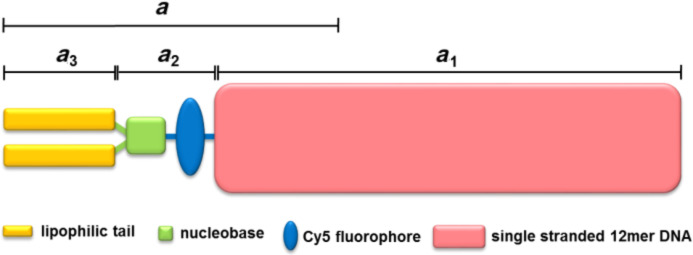
Schematic structure of a Cy5-labeled lipo-oligonucleotide.

Figure 16 schematically displays the structure of a Cy5-labeled lipo-oligonucleotide for the calculation of the semi-major axis *a* according to Equation 1. According to the Stockes*–*Einstein relation for ellipsoidal particles, the diffusion coefficient, *D*, can be expressed as

[1]
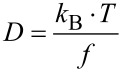


where *k*_B_*T* is the thermal energy, *f* is the frictional coefficient of an ellipsoid, moving at random and η (Equation 2) is the solvent viscosity.

Table 3 summarizes the results of the theoretically calculated hydrodynamic properties of the lipo-oligonucleotides **10–15**. The mean diffusion coefficient was found to be 1.2·10^−6^ ± 0.08 cm^2^ s^−1^.

**Table 3:** Hydrodynamic properties of the lipo-oligonucleotides **10**–**15**.

LON	Nucleo-lipid head group^a^	MW_LON_ [g·mol^−1^]	*a*_1_ DNA length [Å]	*a*_2_ Cy5-base distance [Å]	*a*_3_ lipid length [Å]	2·*b*_anh_ DNA diameter [Å]	*A* Semi-major axis [Å]^b^	*b*_hyd_ Semi-minor axes [Å]^c^	*f*_calc_^d^ × 10^−8^ [g·s^−1^]	*D*_calc_^e^ × 10^−6^ [cm^2^·s^−1^]

**10**	**4a** (11)^a^	4637.8	33.17	13.85	7.35	18.55	27.19	12.08	3.41	1.19
**11**	**5a** (35)^a^	4974.4	33.13	13.53	22.13	18.15	34.40	11.87	3.69	1.10
**12**	**6a** (36)^a^	5054.4	34.48	22.33	25.85	19.00	41.33	12.30	4.09	0.99
**13**	**7a** (19)^a^	4767.8	33.07	13.51	11.85	18.45	29.22	12.02	3.48	1.16
**14**	**8a** (20)^a^	4875.9	32.92	18.78	21.23	18.37	36.47	11.98	3.81	1.06
**15**	**9a** (15)^a^	4707.6	33.68	13.59	6.31	18.29	26.49	11.94	3.36	1.20

^a^Number of C-atoms of the lipid moieties. ^b^From an approximation of an ellipsoid, moving at random [20]. ^c^*b*_hyd_, semi-major axis of hydrated ssDNA. ^d^Calculated viscous drag coefficient of an ellipsoid, moving at random [20]. ^e^Calculated diffusion coefficient.

We do not exclude the formation of LON aggregates, but under our experimental conditions with respect to concentration and temperature, we do not observe a strong aggregation tendency – a finding corroborated by the agreement between the calculated and measured hydrodynamic properties. Any aggregates are obviously not bound within the lipid bilayer, but only in free solution. They act to resupply LONs for the incorporation of LON monomers into the lipid bilayer (Figure 9) by disintegration.

#### Fluorescence correlation spectroscopy (FCS) measurements and experimental determination of hydrodynamic properties

Using fluorescence correlation spectroscopy (FCS), we monitored the diffusion of Cy5-labeled lipophilized oligonucleotides **10**–**15** in aqueous solution within a confocal detection volume of 0.9 fL. The diffusion time, *τ*_D_ (Equation 3), depends on the mass and the shape of the molecule as well as on the dimensions of the detection focus ω_x,y_ in the x,y-dimension, in addition to the diffusion coefficient *D* as

[3]
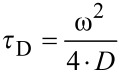


Assuming a diffusion coefficient of *D* = 4.3 × 10^−6^ cm^2^ s^−1^ for MR121 [21–22] and a measured diffusion time of 60 ± 2.05 µs, we calculated the diffusion coefficients for the LONs to be 1.61 ± 0.06 × 10^−6^ cm^2^ s^−1^ (Table 4). This means that the ratio between the experimental and the calculated diffusion coefficients (Table 5, last column) is 1.45 ± 0.09 on average. Our data are comparable with those reported by Fernandes et al. in 2002 [23], which was also determined for DNA 12mers (1.22 and 1.45 × 10^−6^ cm^2^ s^−1^).

**Table 4:** Experimental hydrodynamic data from FCS measurements.

LON	Nucleolipid headgroup^a^	τ_D_ [ms]^b^	χ^2^ [ms]^c^	*D*_exp_ × 10^−6^ [cm^2^·s^−1^]^d^	*D*_exp_/*D*_calc_^e^

**10**	**4a** (11)^a^	0.16 ± 0.007	0.12	1.62	1.37
**11**	**5a** (35)^a^	0.17 ± 0.015	0.27	1.54	1.41
**12**	**6a** (36)^a^	0.16 ± 0.005	0.23	1.62	1.61
**13**	**7a** (19)^a^	0.16 ± 0.006	0.16	1.63	1.40
**14**	**8a** (20)^a^	0.16 ± 0.010	0.28	1.57	1.48
**15**	**9a** (15)^a^	0.15 ± 0.011	0.14	1.68	1.42

^a^Number of C-atoms of the lipid moieties. ^b^Measured diffusion time [ms]. ^c^χ^2^: indicates the quality of the dataset. ^d^Diffusion coefficient calculated from measured diffusion time, *τ*_D_. ^e^Ratio of the experimentally determined diffusion coefficients and the calculated data.

#### Hydration of the lipo-oligonucleotides

In an aqueous environment, water molecules form a hydration shell around the nucleic acid. Consequently, the surface of the nucleic acid chain is smoothed, and the partial volume of the hydrated molecule (*V*_hyd_) is increased by the volume of the water shell. By determining the *V*_hyd_ of the LONs, one can calculate the degree of hydration. The anhydrous volume, *V*_anh_, can be expressed by Equation 4:

[4]
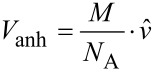


where *M* is the molecular weight of the lipo-oligonucleotide, *N*_A_ is Avogadro’s number, and 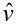 is the partial specific volume.

For the calculation of *V*_anh_ and *V*_hyd_, the volume of the LON is adapted to be an elongated rotation ellipsoid as:

[5]
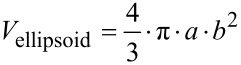


where *b* is the shorter semi-major axis. For the calculations, the parameters *a* and *b*_anh_ and *b*_hyd_ (Table 3), respectively, were used.

The difference *V*_hyd_−*V*_anh_ describes the volume of the hydrodynamically bound water. The degree of hydration is often described as the ratio, δ, of bound water in grams per gram of molecule as described by

[6]
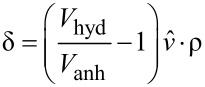


Our calculations demonstrate slight differences between the degree of hydration values depending on the different methods of volume calculation: (i) calculation using *a* or (ii) using *a*_1_/2 (Figure 16). Assuming an increase in the hydrated shell with an increase in the total LONs, the hydration values amount to 0.8–1.2 g of H_2_O per gram of LON. Assuming, however, only hydration of the nucleic acid moiety, the hydration values amount to 0.5 g of H_2_O per g of molecules.

From IR measurements it could be concluded that 20 water molecules hydrate one nucleotide residue within a nucleic acid [24–25]. This results in a hydration of 1 g of H_2_O per gram. However, Bloomfield et al. in 2000 [26] as well as Fernandes et al. in 2002 [23] reported hydration degrees of 0.35 [26] or 0.3 g of water per gram of nucleotide (Table 5).

**Table 5:** *V*_hyd_, *V*_anh_ and partial specific volume 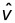 as well as the degree of hydration (δ) for LONs **10**–**15**.

LON	Hydration involving the whole ellipsoid, calculated with major semi-axis *a*					Hydration of DNA strand only, calculated with major semi-axis *a*_1_				
	V*_anh_* × 10^−20^ [cm^3^]	V_hyd_ × 10^−20^ [cm^3^]	V_hyd_−V_anh_ × 10^−21^ [cm^3^]	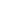 [cm^3^ g^−1^]	δ [cm^3^ g^−1^]	V*_anh_* × 10^−21^ [cm^3^]	V_hyd_ × 10^−20^ [cm^3^]	V_hyd_−V_anh_ × 10^−21^ [cm^3^]	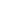 [cm^3^ g^−1^]	δ [cm^3^ g^−1^]

**10**	0.98	1.66	6.81	1.3	0.9	5.97	1.01	4.15	0.8	0.5
**11**	1.19	2.03	8.45	1.4	1.0	5.71	0.98	4.07	0.7	0.5
**12**	1.56	2.62	10.56	1.9	1.2	6.51	1.09	4.41	0.8	0.5
**13**	1.04	1.77	7.28	1.3	0.9	5.89	1.00	4.12	0.7	0.5
**14**	1.29	2.19	9.05	1.6	1.1	5.81	0.99	4.08	0.7	0.5
**15**	0.94	1.60	6.62	1.2	0.8	5.90	1.00	4.16	0.8	0.5

#### Confocal fluorescence lifetime analysis (cFLA)

**Figure 17:**
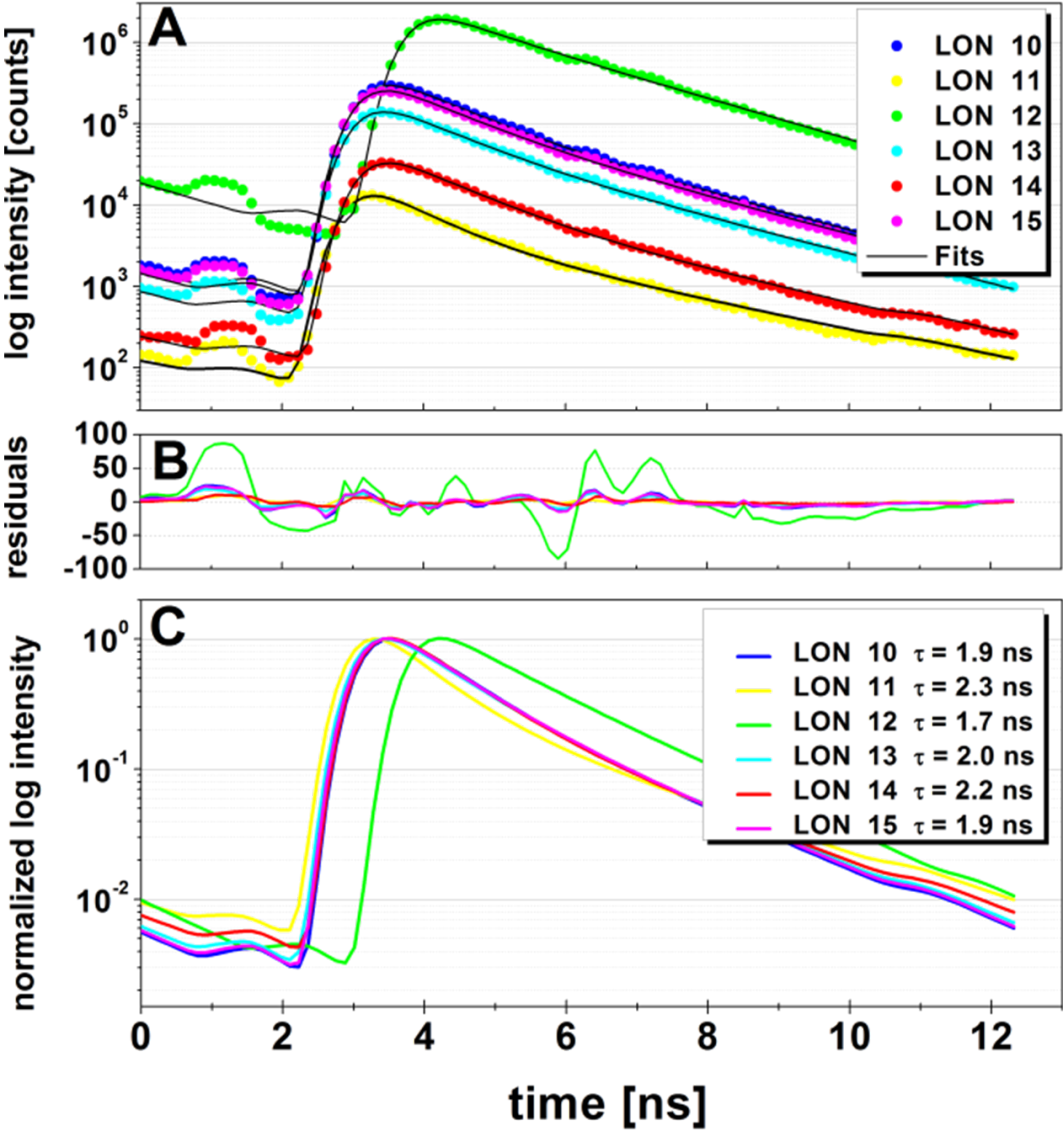
Fluorescence lifetime *τ*_F_ values of the LONs **10**–**15**. A) Logarithmic imaging of fluorescence intensity–time decay of LONs **10**–**15** and the corresponding fitting curves (–). B) The residuals show some minor systematic errors (the color legend is the same as in A and C). C) Normalized logarithmic fluorescence intensity of the fitting curves.

Next, the fluorescence lifetime values of the LONs **10–15** were measured and are listed in Figure 17A–C as well as in Table 6.

**Table 6:** Fluorescence lifetime values of LONs **10**–**15**.

LON	*τ_F_* [ns]

**10**	1.92 ± 0.01
**11**	2.29 ± 0.08
**12**	1.73 ± 0.00
**13**	1.99 ± 0.01
**14**	2.23 ± 0.04
**15**	1.92 ± 0.01

The fluorescence lifetime of the free Cy5 dye is reported to be 0.91 ns [27], 1.0 ns [28–29], and 1.0–1.4 ns [30]. In the literature, fluorescence lifetimes of free Cy5 dye attached to ssDNA as well as to dsDNA, respectively, also exist. The fluorescence lifetime, *τ*_F_, of two 5’-Cy5-ssDNA conjugates were measured as 1.8 ± 0.1 ns [31] for a 36mer and a 23mer [32]. For a Cy5-labeled 25mer, a value of 1.26 ns [27] was reported.

For the LONs **10**–**15** described here, longer lifetime values between 1.7 and 2.3 ns were measured. These data are in accordance with corresponding data of Cy5–ssDNA, and also with those of Cy5–dsDNA.

## Conclusion

The studies described in this manuscript complement the research of various scientific groups whose work is focused on the formation of complex nanoarchitectures [33–34], the interaction of such nanostructures with lipid membranes [35–38], as well as the optimization of the in vivo delivery of lipophilic siRNA [10–12] (e.g., by DNA trafficking [7]).

Further studies from our group will include the binding of terminally lipophilized oligonucleotides at water–air or water–decane boundaries [39] as well as the transdermal application of such LONs through the human Stratum corneum by iontophoresis techniques as well as the transport across the blood–brain barrier [40].

## Experimental

**Materials.** 1-Palmitoyl-2-oleyl-*sn*-glycero-3-phosphoethanolamine (POPE) and 1-palmitoyl-2-oleyl-*sn*-glycero-3-phosphocholine (POPC) were purchased from Avanti Polar Lipids (Alabaster, Al), *n*-decane from Alfa Aesar (D-Karlsruhe), potassium chloride (KCl), 3-morpholinopropane-1-sulfonic acid (MOPS) and 2-amino-2-(hydroxymethyl)propane-1,3-diol (TRIS) were purchased from Roth (D-Karlsruhe). All lipo-oligonucleotides **10–15** (Figure 2) were synthesized, purified and characterized by MALDI–TOF mass spectrometry by Eurogentec SA (B-Liege). In each case, the detected mass confirmed the corresponding calculated mass. MALDI–TOF–MS (*m*/*z*): 4636.8 (**10**, [M + H]^+^; calcd 4637.8); 4976.3 (**11**, [M + H]^+^; calcd 4974.4); 5043.4 (**12**, [M + H]^+^; calcd 5054.4); 4755.0 (**13**, [M + H]^+^; calcd 4767.8); 4866.5 (**14**, [M + H]^+^; calcd 4875.9); 4696.9 (**15**, [M + H]^+^; calcd 4707.6).

Similar to that described in [9,18], we have chosen the particular sequence 5’-d(TAG GTC AAT ACT)-3’, because it does not form hair pins or self-complementary duplexes. The lipophilized strand and the complementary oligomer contain the same number of canonical nucleotides, so that a mixture of the 1 A_260_ units, from both strands, gives an equimolar mixture. For nearly all base pairs, amongst all nearest neighbor combinations exist. Both strands have the same composition; the unmodified duplex exhibits a melting point of 46 °C in a phosphate buffered solution.

**Methods.** The technique of horizontal lipid bilayer fabrication and incorporation of lipo-oligonucleotides is described therein. Analogous to the description in [7–8,41], in the following, the automated bilayer fabrication is described. Horizontal bilayers were automatically fabricated using a lipid mixture of POPE/POPC 8:2 (w/w), 10 mg/mL of *n*-decane within the “bilayer slides” [7–8,41] and observed with an add-on for the inverted confocal microscope (bilayer slides and Ionovation Explorer, Ionovation GmbH, D-Osnabrück). A bent Hamilton syringe (CH-Bonaduz) was used to fill the bilayer slide with 250 mM KCl, 10 mM MOPS/Tris, pH 7 buffer and 0.2 µL of the lipid mixture. The automated bilayer production was started and monitored optically and electrochemically. For the electrochemical measurements, Ag/AgCl electrodes embedded in agarose were used to connect the cis and the trans compartments. As described in previous works [7–8,15,18,41], the measurement protocol is as follows: When a bilayer has been established (C > 50 pF ± 20), a solution of the cyanine-labeled lipophilized oligonucleotide (5 µL, 50 nM, solubilized in water) was injected over the bilayer. The probe was incubated until a stable brightness was reached. During the incubation time, the bilayer integrity was monitored by continuous capacitance measurements. In all graphs, the brightness was given either in kHz or it was normalized (0–100%). The z-direction scan was performed in 5 min intervals (see below). After the incubation steps, the cis compartment was perfused repeatedly for 30 s (1.1 mL/min, each), and the bilayer was inspected by confocal fluorescence microscopy. Each perfusion step using the Ionovation perfusion unit was followed by an incubation step. This procedure was repeated until the brightness of the bilayer stabilized.

All confocal imaging and lifetime measurements were performed on a confocal laser scanning microscope (Insight Cell 3D, Evotec Technologies GmbH, Hamburg, Germany) equipped with a 635 nm emitting laser diode (LDH-P-635, PicoQuant, Berlin, Germany), a 40× water immersion objective (UApo 340, 40×, NA = 1.15, Olympus, Tokyo, Japan), and an Avalanche photodiode detector (SPCM-AQR-13-FC, Perkin-Elmer Optoelectronics, Fremont, CA, USA). Fluorescence irradiation was achieved with an excitation laser power of 200 ± 20 µW directly in front of the objective. Z-direction scans were performed by scanning the confocal laser spot in the *X*–*Y* direction with a rotating beam scanner and movement of the objective in the *Z* direction. The movement in all directions was piezo-controlled, which allows for nanometer precision positioning.

As in [7], each measurement protocol was carried out as follows: (a) a reference scan of the stable bilayer was performed, (b) the lipophilized oligonucleotide sample was added, (c) the solution was incubated until the brightness in the bilayer reached a maximum, (d) followed by an additional series of scans performed after each perfusion of the cis compartment (for 30 s, 1.1 mL/min), and (e) followed by a given incubation time.

In order to analyze the 2D images they were processed with Image J A 1.44 software and the obtained data was evaluated with OriginPro 8 (OriginLab Corporation). For an evaluation of the z-direction scan data, the brightness within the bilayer was measured. The mean area of the bilayer cross section was 400 ± 115 counts per pixel (*N* = 230). Next, the brightness was determined by summing up the number of pixels, where one pixel equals 1 kHz or 1 µm.

All devices used as well as the general techniques used in this paper have been described in detail in preceding manuscripts as well as in an mp4 video [18].
